# Reducing the Shared Burden of Chronic Conditions among Persons Aging with Disability and Older Adults in the United States through Bridging Aging and Disability

**DOI:** 10.3390/healthcare5030056

**Published:** 2017-09-12

**Authors:** Margaret L. Campbell, Michelle Putnam

**Affiliations:** 1 Campbell & Associates Consulting, Seattle, WA 98108, USA; Margaret.Campbell@cjenterprise.net; 2 School of Social Work, Simmons College, Boston, MA 01602, USA

**Keywords:** aging with disability, chronic conditions, evidence-based practice, bridging

## Abstract

Persons aging with long-term disabilities such as spinal cord injury or multiple sclerosis and older adults share similar chronic conditions in mid and later life in the United States. The rising general interest and more prevalent federal requirements for use of evidence-based practices (EBP) in health promotion and chronic condition interventions highlight the gap between demand and the availability of EBPs for persons aging with disability in particular. Addressing this gap will require focused efforts that will benefit substantially by bridging the fields of aging and disability/rehabilitation to develop new EBPs, translate existing EBPs across populations, and borrow best practices across fields where there are few current EBPs. Understanding distinctions between disability-related secondary conditions and age-related chronic conditions is a first step in identifying shared conditions that are important to address for both mid-life and older adults with disabilities. This review articulates these distinctions, describes shared conditions, and discusses the current lack of EBPs for both populations. It also provides recommendations for bridging activities in the United States by researchers, professionals, and consumer advocates. We argue that these can more efficiently move research and practice than if activities were undertaken separately in each field (aging and disability/rehabilitation).

## 1. Changing Demographics of Aging and Disability

As is well established, the proportion of older adults in the global population is rapidly increasing. According to data from the United Nations, between 2015 and 2030 the number of older persons (those aged 60 years or over) in the world is projected to grow by 56%, from 901 million to more than 1.4 billion. By 2030, older persons are projected to outnumber children and to account for 1-in-6 people worldwide, an increase from 1-in-8 people in 2015. Additionally, life expectancy has increased worldwide—60% of women and 52% of men born between 2000 and 2005 are expected to survive to their 80th birthdays, compared to less than 40% of the women and men born between 1950 and 1955 [1]. In relation to disability, according to the World Health Organization’s (WHO) World Report on Disability, about 15% of the world’s population (or 1 billion people) currently lives with some form of disability, and between 2% and 4% experience severe disability [2]. Based on WHO projections in the same report, the number of people with disabilities is growing and expected to continue to grow, due to both population aging and the global increase in chronic health conditions associated with disability, such as diabetes, cardiovascular diseases, and mental illness. In sum, having a disability places a person in one of the world’s largest minority groups.

Over the past several decades, the increased longevity among people with disabilities has emerged as an important demographic trend to consider in regards to health promotion and chronic conditions. This phenomenon of increased longevity is often described as *Aging with Disability* [3,4,5]. While there is no empirical standard for defining who is *Aging with Disability*, the general agreement is that the term refers to people living with the long-term effects of disabilities acquired from birth to middle age who are now surviving into mid- and later life [6,7,8,9]. People who are aging with a disability include traditional rehabilitation populations (e.g., people with spina bifida, cerebral palsy, neuromuscular disorders, polio, spinal cord injury, traumatic brain injury, multiple sclerosis, etc.), and persons with intellectual and developmental disabilities.

The size of the aging of the disability population worldwide or for specific nations is largely unknown. Some national estimates are available, but they tend to be imprecise due to differing ways disability is defined and measured (e.g., functional limitation, disease diagnosis, inability to work) as well as incomplete data often related to the omission of questions on age of onset and/or the duration of primary disability [10]. The most recent inference for the United States (U.S.) suggests that between 12 and 13 million adults 18 years and older are living with an activity limitation acquired before age 40 [11,12]. This is a small but significant sub-set of the overall population of persons with disabilities living in the U.S., estimated to be 39.9 million in 2014 based on a self-reported functional limitation, or 12.6% of the community of the living population over 5 years of age [13]. It is an unknown percentage of the older adult population aged 65 or older, which in 2014 was estimated to be 46 million or 14.9% of the total U.S. population [14]. While the aging with disability population may seem modest in comparison to these larger groups, the longevity trends of this sub-population represent significant advances made in medicine, rehabilitation, education, social and employment programs, and disability rights, access, and inclusion over the past fifty years. 

This paper focuses on persons aging with disability in the U.S as a case example. It articulates distinctions between disability-related secondary conditions and age-related chronic conditions as a first step in identifying shared conditions important to address for both mid-life and older adults with disabilities. It also provides recommendations for bridging activities in the United States by researchers, professionals, and consumer advocates. We argue that these can more efficiently move research and practice than if activities were undertaken separately in each field (aging and disability/rehabilitation).

## 2. Clarifying Understanding of Aging with Disability and Chronic Conditions

In general people aging with long-term disability with onset prior to later life in the United States are concentrated among the population ages 40-to-64 [7]. In our review of the literature, we were unable to identify similar data for other nations; however, we recognize there may be differences in disability trends based on wealth and income patterns, as well as the global burden of disease [15] This population is distinguished from people aging into disability in later life and those who acquire sustained significant disability for the first time at older ages [8].

**Figure 1:**
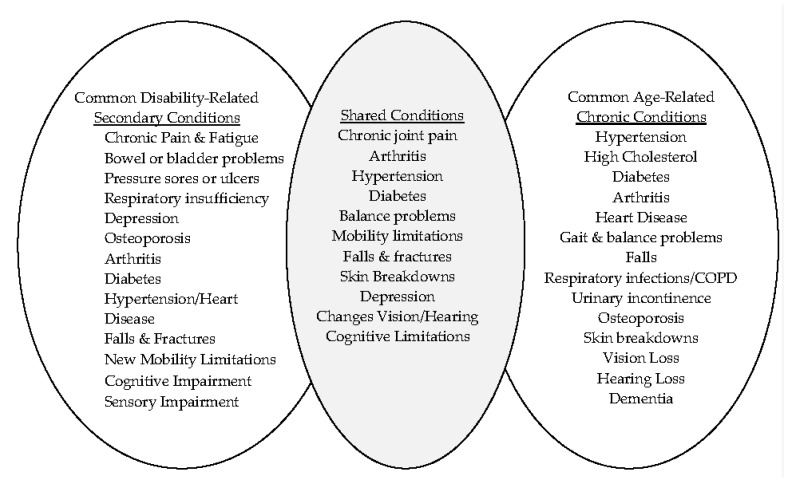
Examples of shared chronic conditions of persons aging with disability and aging into disability in later life.

It is clear from the research literature that people with an early onset of disability, beginning at approximately age 45, report greater difficulty with independent living than they did at early onset. A challenge clinicians, practitioners, and individuals aging with disabilities themselves encounter in working with persons aging with long-term disability is distinguishing the effects of living with long-term disability from the onset of age-related secondary conditions. Difficulty in making this distinction stems from (1) a lack of clear terminology in clinical practice and research; and (2) the need to disentangle the concepts of secondary conditions, related to a primary disability, and chronic conditions, which are generally not related to the primary disability, but to aging, so that they are not mistaken for each other or conflated. We present a brief distinction between these concepts and show overlap in shared conditions in Figure 1 below.

### 2.1. Disability-Related Secondary Conditions

Research and clinical observations, together, show that as individuals aging with disabilities continue to live longer there is an increased likelihood of experiencing secondary conditions directly or indirectly. These secondary conditions are typically related to the underlying disability, including the onset of new functional changes and declines, and can threaten independence and participation and lead to secondary disabilities [16]. Secondary conditions associated with disability are defined as any additional physical or mental health conditions that can result from a primary disabling condition but are not a diagnostic feature of that condition [17]. This includes preventable physical, mental, and social disorders resulting directly or indirectly from an initial disabling condition [18] that are generally considered direct secondary conditions. It is not unusual for indirect secondary conditions to be experienced by people living with long-term disability. Indirect secondary conditions are in part due to physical overuse of compensatory limbs (e.g., shoulder injuries for manual wheelchair uses), sedentary life styles, environmental barriers to recreational and leisure opportunities, and poor health behaviors. These are frequently the same as the age-related chronic conditions experienced by the aging population at large [19,20].

While precise data are not available for the U.S. population, clinical and survey research indicate that the high rates of complications and/or changes experienced by people with long-term disability typically occur about 20–25 years sooner compared to peers without disabilities [5]. Specifically, “… as persons with long-term disability reach age 50, many show the kind of functional ages that would *not* be expected until age 70–75 in people without disabilities” [21]. Given the timing of the onset of aging with disability, typically starting in the mid-40s, and the impact on physical function, secondary conditions have been described in the literature as evidence of *pre-mature, atypical, and accelerated aging* [5,8]. While the onset and severity of secondary conditions varies by the severity and duration of the underlying disability, research conducted during the last 25 years indicates that the most common conditions include the following: chronic pain and fatigue, bowel or bladder problems, pressure sores or ulcers, respiratory insufficiency, depression, osteoporosis, accelerated arthritis, falls & fractures, new mobility problems, hypertension, heart disease, and sensory and cognitive impairments [8,18,22].

### 2.2. Age-Related Chronic Conditions

Current trends in longevity and population growth are associated with the rise in the prevalence of chronic disease and other chronic conditions and combinations of chronic conditions associated with aging and growing older, long-term effects of exposure to environmental hazards, and long-term effects of poor health behaviors or the practice of poor health behaviors over time. Although the literature does not support a single uniform definition for chronic conditions, recurrent themes or criteria include: the non-self-limited nature of the condition, requiring ongoing medical attention; the association with persistent and recurring health problems and diseases, and activity limitations; and a duration measured in months and years, not days and weeks. Chronic conditions include both *physical conditions,* such as arthritis, sensory impairments, cancer, and HIV infection, *and cognitive disorders*, such as ongoing depression, substance abuse, and dementia [23,24]. Examples of common age-related chronic conditions include: hypertension, high cholesterol, diabetes, osteoarthritis, heart disease, gait and mobility problems, falls, respiratory infections/chronic obstructive pulmonary disease (COPD), urinary incontinence, osteoporosis, skin breakdowns, hearing and vision loss, and dementia. In the United States, the onset for chronic conditions and diseases begins in midlife for just over half of all adults. Roughly 53% of adults with chronic conditions ages 45–64 have at least one chronic condition and nearly 86% of adults age 65 and older have at least one chronic condition [25]; and thus, they are generally described as *age-related chronic conditions* [23]. 

### 2.3. Multiple Chronic Conditions

Similar to the risk of *secondary disabilities* for people aging with long-term disability, multiple chronic conditions (MCCs) are concurrent chronic conditions that affect a person at the same time [23,26]. The most common MCC dyads are hypertension and arthritis, hypertension and high cholesterol, and hypertension and diabetes. The most common MCC triads are hypertension, high cholesterol, and diabetes, and hypertension, high cholesterol, and arthritis [26]. The effects of CCs and MCCs on individuals, families, and healthcare systems are many. For individuals, CCs and MCCs can negatively affect physical and psychosocial functioning, independence, and participation in community life, including work. CCs and MCCs are associated with a greater risk of poor day-to-day functioning. In addition, they can diminish quality of life and add to the demands on family members or others as caregivers. In some cases, MCCs may be simply a cluster of aging-related chronic conditions found among older individuals, but not all MCCs are age-related. Some individuals may experience MCCs at younger ages such as hypertension and high cholesterol. U.S. data show that just over 7% of adults ages 18–44 have MCCs (19% have a single CC) [25]. The common CCs and MCCs noted above may also include age-related CCs not commonly found among younger adults such as dementia, urinary incontinence, and gait problems for persons aging with and aging into disability.

At a systems level, CCs and MCCs can challenge health care professionals whose education has not prepared them to care knowledgeably for people aging with long-standing disabilities. CCs and MCCs increase the risk for repeat hospitalizations and of receiving conflicting advice from physicians and other health care providers. They also contribute to frailty, functional limitations, and disability, which further complicate access to health care, interfere with self-management, and necessitate reliance on caregivers. In general, MCCs are associated with substantial health care costs: in the U.S., about 66% percent of the total health care spending is related to care for CCs/MCCs [23].

### 2.4. Understanding the Nexus of Aging and Disability

As Figure 1 demonstrates, persons aging with disability and older adults share a common set of chronic conditions as a result of experiencing disability-related secondary conditions, particularly indirect secondary conditions, or age-related chronic conditions. In addition, persons aging with disability may also experience age-related chronic conditions as well as disability-related secondary conditions, making the intersection of shared conditions wider. Based on disease diagnosis or impairment status, there may be some variances between persons aging with disability and older adults in onset, the specific nature and/or symptoms related to the conditions, or in the way these conditions interact with primary disability or other chronic conditions. That said, there is arguably more potential to advance clinical and program interventions that address these shared chronic conditions through research that develops more universal interventions that have efficacy for both persons aging with disability and the traditional older adult population than by pursuing two distinct lines of intervention research and practice (one for each population group). In sum, segmenting *people aging with disabilities* from *people aging into disability in later life*, those who acquire a sustained disability for the first time at older ages [9] (we place persons with CCs/MCCs in this latter group), can be considered inefficient. It would be more relevant to work towards a better understanding of the nexus of aging and disability [27], focusing on shared chronic conditions, so that more universal interventions can be developed.

## 3. The Shared Burden of Chronic Conditions

Historically, there has been little focus on the shared burden of chronic conditions across persons aging with disability and older adults in CC practice and research. This may be due to the age targeting of public health research and program interventions or, in some cases, efforts aimed at populations with specific disease and/or diagnostic conditions. The chronic conditions evidence base tends to straddle two substantially separate literatures, gerontology and rehabilitation/physical medicine, which typically focus on older and younger/middle-aged adults, respectively. Public health literature is less age-specific, but it remains rare to find research that attempts to do any of these three things which would help build the evidence base: (1) understand disability and chronic conditions across the life course; (2) include both persons aging with and aging into disability; and (3) attempts to translate evidence, including interventions, from younger persons with disabilities to older persons with disabilities and vice versa. It is also rare to find practice guidelines with any of the above traits. In sum, this approach of segmenting the study of chronic conditions by age group and disability history or diagnosis has substantially limited our understanding of chronic conditions among persons aging with a disability in particular, creating a shortage of evidence-based practices to promote health and reduce the burden of chronic conditions. For example, to date, there are few empirical studies of aging with disability for midlife and older persons in general [8,28,29]. Table 1 presents indicators of the nexus of aging and disability in the U.S., highlighting the shared burden of CCs.

## 4. The Growing Demand for More Evidence-Based Practices (EBP) to Promote Health and Reduce the Shared Burden of Chronic Conditions in the U.S.

There has been agreement across aging and disability sectors, and within U.S. federal agencies focused on health, aging, and disability, that prevention of chronic conditions should be a major component of health promotion for older adults and people with disabilities [34]. In response to the high rates of chronic conditions and diseases observed for middle-aged and older adults with and without disabilities, between 2000 and 2016, the U.S. Congress passed legislation establishing new federal programs and modified administrative regulations to require and encourage the delivery of evidence-based (EB) health promotion and disease prevention programs, as well as services for older adults and populations with greater likelihood of experiencing chronic conditions, including persons with disabilities and individuals within racial and ethnic minority groups. Examples of key legislation include the 2010 Patient Protection and Affordable Care Act (ACA) Title IV: Prevention of Chronic Disease and Improving Public Health. While the primary focus of Title IV is on removing financial barriers and increasing access to preventative healthcare services, it also awards competitive grants to eligible entities for programs that improve health, reduce chronic disease rates, address health disparities, and develop stronger evidence based on effective prevention programming. While there are ongoing calls for revisions and the repeal of the ACA, which may reduce funding or eliminate these programs, the costs of chronic disease at the state and federal level are well documented [35] and may be persuasive in continuing the emphasis on the health promotion program regardless of any political discord.

There are other legislative and administrative regulation changes, which also support greater use of EBPs that may be more politically durable. For example, beginning in 2016, all funds for disease prevention and health promotion services delivered under the Older Americans Act Title IIID and administered by the Administration on Aging (AoA) within the U.S. Administration for Community Living (ACL) may only be spent on programs that meet the highest-level criteria for evidence-based practices (e.g., use of experimental and quasi-experimental research designs and translation into community settings per a new administrative rule) [36]. Another example is the U.S. Administration on Community Living (ACL) itself, which receives funding from various legislated programs including the ACA, the OAA, and the Rehabilitation Act to achieve its mission to “maximize the independence, well-being, and health of older adults and people with disabilities across the lifespan” [37]. Key health promotion programs under the ACL include Disease Prevention and Health Promotion Services (DPHPS), Aging and Disability Network of Evidence-Based Programs (ADEPP), and Chronic Disease Self-Management Education Programs (CDSMP). All of these programs support the development of and implementation of evidence-based programs.

Since the change in presidential administration in January 2017, it is not clear what the future of these legislative programs and their administration will be as most reflect the priorities of the prior presidential administration. However, the costs of treating chronic conditions and the premature deaths related to them are well established [26,38,39] and there is substantial interest in programs, interventions, and treatments that bring the best evidence to bear in addressing chronic conditions by other stakeholders including individuals with chronic conditions, practitioners and service providers, and health care organizations that are likely to keep the momentum for the development and implementation of EB health promotion and chronic care intervention programs moving forward. These are positive developments, but they also highlight the substantial gap between the critical need for EBP health promotion programs that have been tested and successfully implemented in community settings (e.g., in real homes, neighborhoods, and community-based organizations) and their supply, which are notably few. While this imbalance between supply and demand exists for all adults with disabilities and activity limitations, the gap is more pronounced for middle aged individuals with early onset disabilities than for their older counterparts. For example, a scoping review of the National Council on Aging (NCOA) Center for Healthy Aging database containing all the health promotion programs that meet the ACL criteria for evidence-based practices found that only 2 of the more than 150 randomized controlled trials listed included middle-aged individuals with early-onset, long-term disabling conditions in the clinical trial of the intervention. The other 148 studies were based exclusively on older adults with and without chronic conditions, but no significant disabilities [18].

Additional evidence of the gap in EB health promotion programs for persons aging with a disability exists. ACL maintains a webpage entitled Aging and Disability Evidence-based Programs and Practices (ADEPP) that lists the EBPs that are ready for dissemination. Of the three EB health promotion programs with the highest level of evidence under the OAA Title IIID criteria (Stanford’s Chronic Disease Self-Management Program (CDSMP), A Matter of Balance for fall prevention, and Health IDEAS for depression management [40]) only the CDSMP has been evaluated for effectiveness with people with disabilities under research conditions. However, it has yet to be implemented for this population in standard practice in community-based settings, so it is unclear how well the intervention will translate to everyday practice. Similarly, of the 12 EB health promotion interventions listed on the ADEPP website [41], which are maintained by the U.S. Administration for Community Living (ACL), none have been tested and demonstrated to be effective for middle aged individuals with an early onset of long-term disabilities. The net result is that despite ACL’s mission, people aging with long-term disabilities between the ages of 45 and 64 are less likely to receive EB health promotion programs in community settings through their programs compared to older adults with and without activity limitations.

In part, the explanation for the shortage in EBP to reduce the burden of chronic conditions for people aging with a disability can be explained by differences in research investment. While both aging and disability fields face the same increased demand for the delivery of effective EB health promotion programs in community settings, the nature of the gap is different. From the aging perspective, there are a sizable number of EBPs related to chronic care and health promotion. The gap results primarily from a lack of funding for translational and implementation science research aimed at adapting the portfolio of existing EB health promotion interventions for older adults for delivery in community-based settings. Whereas, from the aging with disability perspective, the gap is more fundamental and results from both (1) a historical lack of national attention to the health and wellness needs of people with disabilities in general, related in part to outdated stereotypes that disability is synonymous with poor health; and (2) a historical lack of government funding for basic intervention development research for middle-aged adults aging with long-term disabilities, ready to be translated and implemented in community settings, that would create a portfolio of EB health promotion programs for adults with disabilities [42]. It is possible to close this gap, but focused initiatives and actions will be required to do so.

## 5. Closing the EBP Needs and Supply Gap by Bridging Aging and Disability Research and Practice

To help close the gap in our data and knowledge and increase the availability of evidence-based health promotion programs that reduce the burden of chronic conditions experienced by people aging with disability in midlife and beyond, key actions are needed both at the systems and the practice levels that work to bridge the fields of aging and disability. Bridging aging and disability is noted to be the process of actively promoting the development of mechanisms that create pathways across fields of research and practice, foster interdisciplinary collaboration, and pursue a common knowledge base that can support both sharing and shared evidence [43]. Engagement in bridging work has the potential to more efficiently work towards closing the gap between the need for EBPs that reduce the shared burden of chronic conditions and the supply of these programs and interventions. Below we outline several recommendations for where and how to begin this bridging work that we believe have considerable potential for success.

At the systems level, aging, disability, and public health agencies within the government can have a significant impact on the development, implementation, and dissemination of EBPs by taking the following concrete steps. First, advance understanding of the patterns and trajectories as well as increase the ability to study chronic conditions over time by developing and funding new (or providing expanded funding for existing) public longitudinal data systems that include both younger and older individuals aging with long-term disabilities. Rather than setting an ending or beginning point of a data set at age 60 or 65, the sample base should span the transition from mid-life into later life. Age segmenting data sets with cut-offs of age 60 or 65 do not permit an understanding of the aging with disability process and its relationship to chronic conditions. A good example of this is the National Health and Aging Trends Study (NHATS), a new longitudinal data set launched in 2011 [44]. NHATS includes a wide set of bio-psycho-social domains but the age range of the sample is 65 and older. Some U.S. longitudinal data sets do have wider age ranges, e.g., the Panel Study on Income Dynamics (as early as birth) [45] and the Health and Retirement Study (age 50 and older) [46], but few contain measures of the age of onset and the duration of onset of disabilities, as well as the age of onset of significant activity limitations and the duration of activity limitations. This limits the possibilities for identifying persons aging with a long-term disability and the ability to better identify [47] and understand the intersections of age-related chronic conditions and disability-related secondary conditions. That said, it can be argued that data sets that start at age 50 do permit an analysis of persistence of disability over time, regardless of the lack of onset measure [9]. Depending on the purpose of the analysis, this may be accepted. However, as a second action, we recommend that the new and/or expanded data sets include measures of the age of disability diagnosis, the age of disability onset, and other relevant time variables. 

Third, government aging and disability agencies should work in collaboration with private funders, foundations, and affiliated organizations to bring scientists, policy experts, program administrators, and other stakeholders together in organized events to develop a coordinated research agenda that includes both the traditional older population and persons aging with disability. This agenda should identify priorities that respond to the challenges of aging with disability and chronic conditions across the life cycle. At this time, the lack of an agenda hinders strategic, forward progress in creating the supply of EB health promotion programs and chronic condition interventions and limits opportunities to leverage public and private funds. Fourth, in response to this research agenda, government and private sector funders and organizations should consider investing in new joint funding initiatives that support interventions development, efficacy, and effectiveness trials, as well as translational research and scaled up evaluations of evidence-based health promotion programs that include both traditional aging populations and persons aging with a long-term disability. 

Fourth, and finally, government agencies, as well as private funders, should invest in the development of researchers with knowledge and capacity in bridging aging and disability to support work across traditional aging and disability populations. They should also support the development of researchers who have dedicated training related to aging with disability. This can be done by providing funding for cross-disciplinary training of new and seasoned researchers as well as by supporting pre- and post-doctoral fellowships. This will expand the workforce of scientists available to work on EBPs related to health promotion and chronic conditions for adults with disabilities of all ages.

At the practice level, researchers, educators, providers, and consumer advocates can facilitate and support efforts to bridge the aging and disability program and practice networks by doing the following. One, seek and develop partnerships and collaborations that cut across the aging and disability practice and service networks. These partnerships can do many things ranging from helping individuals find resources and support, to providing the resources and platforms necessary to support community-based research, to assisting in the implementation of evidence-based health promotion programs. In the United States, examples of programs where these opportunities for bridge building lie are the Aging and Disability Resource Centers [48] which are charged with helping individuals with disabilities of all ages understand and access support and service options. Without these partnerships and collaborations, it is difficult to share existing knowledge or advance a more unified evidence base. 

Two, where there is a gap in the evidence base, practice professionals can draw on best practices from both the aging and disabilities bodies of knowledge when selecting health promotion programs and chronic care intervention approaches until the gap is filled. In sum, practitioners should look across fields of study and practice, as shared conditions between the older adults and persons aging with disability suggest that within the nexus of aging and disability, it is possible for practices used with one population to benefit the other. 

Three, researchers, educators, providers, and consumer advocates should educate themselves on aging and disability policy, funding streams, and service delivery systems so that they understand more broadly where chronic condition programs are provided, how they are delivered, and how they are funded. This broad knowledge will help guide persons in need of assistance to potential resources, as well as increase awareness of the gap in the provision of chronic condition interventions and health promotion programs. Among other things, this knowledge can be used by professionals and advocates to advocate for funding changes that help bridge aging and disability programs, which have historically segmented clients and consumers by age, often providing different sets of benefits to different age and/or diagnostic or impairment groups.

Finally, four, researchers, educators, providers, and consumer advocates should actively advocate for the rights of people aging with disabilities and older adults aging into disabilities to receive effective, evidence-based health promotion programs in community settings. This highlights a true bridge between the fields of aging and disability—the desire to live in the community—that has the potential to overcome historical differences between aging and disability sectors [49]. In this pursuit, professionals and consumer advocates would be well-served to use inclusive language that reflects a bi-cultural understanding of the similarities and differences in the aging and disability experience, honoring the history and values of the respective aging and disability movements, in an effort to advance shared interests.

## 6. Conclusions—Reducing the Shared Burden of Chronic Conditions

In the U.S., the divide between aging and rehabilitation/physical medicine has left a substantial void in our understanding of how best to intervene in real-life settings to improve health by addressing secondary conditions related to aging and age-related secondary conditions. However this void is visible internationally as well, as evidenced in the academic literature. The U.S. is just one case example. The growth of the aging with disability population is a worldwide phenomenon that is under-studied, including the role of CC/MCC’s in exacerbating disability and increasing premature mortality. As increased global attention is paid to non-communicable diseases by the World Health Organization and other leading international non-governmental organizations [50], there is a need to consider the most effective and efficient ways of developing and implementing health promotion interventions for persons with disabilities of all ages. The increased longevity experienced by both middle-aged and older adults living with long-term disabilities and significant activity limitations, combined with the high risk of secondary and chronic health conditions, reinforces the importance of bridging knowledge, policy, and practice across aging and disability fields to better serve the emerging population of persons aging with disabilities. Recognizing the shared burden of chronic conditions and taking steps to reduce the burden as efficiently as possible by recognizing this shared interest is critical to closing the gap in the evidence base and developing community-based, health-promotion programs and chronic condition interventions that are readily usable and benefit all adults aging with disabilities.

## Figures and Tables

**Table 1:** Indicators of the nexus of aging and disability in the U.S.

*Overall disability prevalence is substantial.* In 2014, 39.9 million or 12.6% of the community of the living population ≥5 years or older reported having a disability [25].
*Disability prevalence is increasing over time.* The total number of non-institutionalized civilians ≥age 18 reporting disability increased by 2 million between 2005 and 2010 [30].
*Prevalence of disability increases with age.* Disability rates are 10.7% for persons age 21–64 years; 25.4% for age 65–74 years; and 49.8% for ages 75 and over [31].
*Prevalence of multiple disabilities increases with age.* Prevalence of three or more disabilities are 18.2% for persons ages 18–44 years; 27.6% for age 45–79 years; and 45.4% for ages 80 and older. Mobility disabilities are most likely to co-occur with other types of disabilities. For example: among persons who report movement difficulties, 27.6% also report sensory difficulties and 42% reported work limitations [7].
*Persons with early and mid-life significant disability have increased life expectancy.* Research studies indicate that people with physical disabilities now have nearly normal life spans [30,31]. People with developmental disabilities now routinely live beyond 50 [32].
*Persons with existing disabilities experience increased disability over time* [7]. Between 1998 and 2011, chronic disability rates increased significantly across groups of individuals with six different types of conditions (movement, emotional, cognitive, self-care, social & work) among civilian, non-institutionalized adults over 18, and older based on data from the NHIS. The greatest increase was for movement difficulties, which increased from 19.3 to 23.3% in persons 18 and older [33].
*Substantial percentages of individuals report the average age of onset of primary disability as before age 65.* 30% of individuals with disabilities report experiencing onset at age 44 or younger. 13% of 15–64 year olds reported disability onset at birth. Half of all persons age 65 or older reported having activity limitations before age 65 [26].
